# Performance Evaluation of ChaosFortress Lightweight Cryptographic Algorithm for Data Security in Water and Other Utility Management

**DOI:** 10.3390/s25165103

**Published:** 2025-08-17

**Authors:** Rohit Raphael, Ranjan Sarukkalige, Sridharakumar Narasimhan, Himanshu Agrawal

**Affiliations:** 1 School of Electrical Engineering, Computing and Mathematical Sciences, Curtin University, Perth 6102, Australia; himanshu.agrawal@curtin.edu.au; 2 Department of Chemical Engineering, Indian Institute of Technology Madras, Chennai 600036, India; sridharkrn@iitm.ac.in; 3 School of Civil and Mechanical Engineering, Curtin University, Perth 6102, Australia; p.sarukkalige@curtin.edu.au; 4 Wadhwani School of Data Science and Artificial Intelligence, Indian Institute of Technology Madras, Chennai 600036, India

**Keywords:** IoT, cryptography, WDN, WSN, LoRa, Arduino, data security

## Abstract

The Internet of Things (IoT) has become an integral part of today’s smart and digitally connected world. IoT devices and technologies now connect almost every aspect of daily life, generating, storing, and analysing vast amounts of data. One important use of IoT is in utility management, where essential services such as water are supplied through IoT-enabled infrastructure to ensure fair, efficient, and sustainable delivery. The large volumes of data produced by water distribution networks must be safeguarded against manipulation, theft, and other malicious activities. Incidents such as the Queensland user data breach (2020–21), the Oldsmar water treatment plant attack (2021), and the Texas water system overflow (2024) show that attacks on water treatment plants, distribution networks, and supply infrastructure are common in Australia and worldwide, often due to inadequate security measures and limited technical resources. Lightweight cryptographic algorithms are particularly valuable in this context, as they are well-suited for resource-constrained hardware commonly used in IoT systems. This study focuses on the in-house developed ChaosFortress lightweight cryptographic algorithm, comparing its performance with other widely used lightweight cryptographic algorithms. The evaluation and comparative testing used an Arduino and a LoRa-based transmitter/receiver pair, along with the NIST Statistical Test Suite (STS). These tests assessed the performance of ChaosFortress against popular lightweight cryptographic algorithms, including ACORN, Ascon, ChaChaPoly, Speck, tinyAES, and tinyECC. ChaosFortress was equal in performance to the other algorithms in overall memory management but outperformed five of the six in execution speed. ChaosFortress achieved the quickest transmission time and topped the NIST STS results, highlighting its strong suitability for IoT applications.

## 1. Introduction

Recent technological advancements have exerted a significant influence on daily life. Innovations aimed at improving accuracy, speed, and efficiency are inducing both qualitative and quantitative transformations in prevalent operational systems [1]. The Internet of Things (IoT) is an integral technological tool that facilitates advanced sensing, actuation, communication, and control among a network of interconnected physical devices. This integration enables significant improvements in automation and operational efficiency across numerous domains, thereby contributing to an enhanced quality of life [2]. This adoption of IoT has seen an exponential increase with the development of smart sensors and devices [3]. The increasing volume and complexity of data necessitate specialised tools for effective management. This requirement is particularly evident in the diverse applications of the Internet of Things (IoT) across numerous sectors, including smart cities, utility management, healthcare, commerce, and environmental monitoring.

Water distribution networks (WDNs) represent a critical component of public utility management. The sustainable and equitable monitoring, supply, and maintenance of water for all end-users necessitate robust operational oversight. Consequently, these extensive systems generate vast quantities of data, for which effective management is important. The secure collection, transmission, and storage of this information are essential to maintain data integrity, prevent fraudulent activities, and mitigate significant public safety risks. Given that the operational data of WDNs has historically been a target for malicious attacks, the implementation of advanced security protocols is a critical priority. Hassanzadeh et al. analysed 15 data security incidents spanning 18 years in the water sector [4], emphasising the rise in ransomware, data theft, inside threats and attack complexity. SunWater Breach [5] in Queensland lasted undetected for nine months when attackers exploited outdated infrastructure to access the customer info web server. In February 2021, a remote attacker tried to raise the sodium hydroxide levels of the Oldsmar water treatment plant from 100 ppm to 11,000 ppm, which could have led to a city-wide poisoning [6]. In January 2024, another remote attack triggered a water system overflow in Texas [7]. The officials had to unplug the system and operate it manually, while they had 37,000 attacks on the system in four days. Following similar incidents, the officials of the US Environmental Protection Agency gave formal suggestions pointing out the higher probability of drinking water and wastewater systems becoming targets of attackers, due to a lack of resources and technical capacity to adopt cybersecurity measures. The concept of data security in cryptographic algorithms ensures the confidentiality and integrity of data. The large amount of data generated from the associated devices increases the susceptibility to attacks [8,9]. Traditional cryptographic algorithms demand heavy resources in computation and power, which is difficult for IoT devices designed with a minimalist approach in terms of size, computational power, energy usage, and memory requirements. Thus, there is a need for a lightweight solution that is suitable for devices running on simple microcontrollers [10]. This also raises the question of the compatibility of the algorithms to be more suitable for a chip-level implementation than a software application [2,9].

Data security becomes challenging when the data flow involves multiple communication interfaces from local wireless networks to the Internet. The communication of data from the edge devices to a central gateway or cloud-based data management platform makes them susceptible to undesired attacks like denial of service, man-in-the-middle, zero-day, identity theft and malware [9]. Thus, having a functional, lightweight, and effective encryption system through cryptographic algorithms is necessary to operate any IoT sensor network.

**Figure 1:**
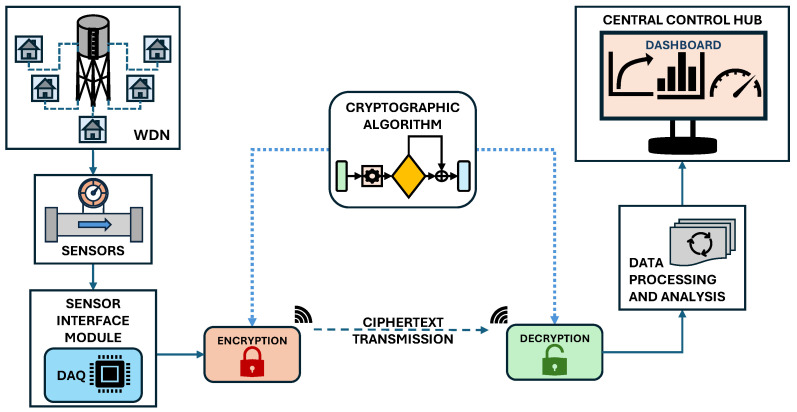
Implementation of cryptography in water distribution network.

In a utility management context, the implementation of cryptographic algorithms is essential for securing wireless sensor data communication. The process of applying a cryptographic algorithm to a water distribution network is illustrated in Figure 1. Data collected from various sensors, such as flow, level, and pressure sensors, is first gathered by a sensor interface module, which functions as a data acquisition unit. This module is equipped with data conditioning capabilities, which are used to encrypt the data before transmission. The original plaintext data is transformed into a ciphertext through a cryptographic conversion and then transmitted. Upon reception, the ciphertext is converted back into the original data through a decryption process, which uses the same cryptographic algorithm. The decrypted data is subsequently analysed and transferred to a central control hub responsible for data storage, further analysis, and visualisation.

Lightweight cryptography is specifically tailored for resource-constrained devices, the proliferation of which has been driven by the widespread adoption of portable electronics. Lightweight cryptography resulted from a project started by the National Institute of Standards and Technology (NIST) in 2013 to explore applying NIST-approved cryptographic tools on constrained devices in IoT applications. This study compared performance and resources regarding energy consumption, latency, and throughput. While conventional cryptography focuses on devices like personal computers, servers, smartphones, and tablets, lightweight cryptography targets resource-constrained devices like embedded systems, RFID, and sensor networks [11].

The existing cryptographic algorithms face certain challenges that are addressed in the ChaosFortress algorithm. Our contributions are as follows.

- Faster performance in the execution of the cryptographic operations and transmission of the payload is one of the key highlights of the ChaosFortress library. The execution time in encryption and decryption operations is second only to the Speck algorithm. However, the deterministic nature of the Speck algorithm, which results in a lack of cipher randomisation, renders it less secure than ChaosFortress [12,13]. In contrast, ChaosFortress is non-deterministic, generating a unique ciphertext for identical plaintext inputs over successive encryption cycles. The non-deterministic nature also helps ChaosFortress block ciphertext-only attacks and replay attacks. Although the encryption operation inside the ChaosFortress library is done through bitwise operations, the final stage of the algorithm implements a HEX conversion, which makes the payload lighter and easier to transmit. ChaosFortress performed the fastest among the tested algorithms in transmission tests, with the next best performer, ACORN, performing three times slower than ChaosFortress.
- Float input optimisation injected into the ChaosFortress algorithm is optimised for sensor data, which mostly contains floating point values. This avoids preconditioning the data using scaling when the original data has fractions or decimals.
- Security features integrated into the ChaosFortress algorithm, even while using a lightweight design, are evident from the NIST STS performance of ChaosFortress, with 98.1% in the pass percentage in the statistical tests. This is crucial in ensuring smooth operation in resource-constrained devices without compromising security. The security analysis is crucial for evaluating the effectiveness of a cryptographic algorithm. NIST STS Statistical Test Suite, developed by the National Institute of Standards and Technology, examines the randomness of binary sequences generated by random or pseudorandom number generators within cryptographic contexts. The strength of the lightweight cryptographic algorithms depends heavily on the ability to produce statistically random outputs, which can be tested using NIST STS [14].

## 2. Related Works

Lightweight cryptography has been of interest since the onset of low-power IoT devices, designed to manage simple applications in a smart environment. Multiple studies and technologies have been introduced over the past years, supporting the necessity of lightweight cryptographic systems. Koteshwara and Das [15] discussed using the Authenticated Encryption with Associated Data (AEAD) scheme targeting lightweight IoT applications. This study offers practical recommendations for selecting AEAD schemes for embedded and IoT devices. Yasmin and Gupta [16] proposed a modified GIFT block cipher in the field of 3D printer security, analysing its effectiveness on six parameters, namely computation time, power, energy, latency, throughput, and software efficiency. GIFT improves applications’ security, efficiency, and hardware footprint in resource-constrained environments. Panahi et al. [17] assessed ten lightweight block ciphers and evaluated their performance on Raspberry Pi 3 and Arduino Mega 2560, which are popular low-power devices used for IoT applications. This is highly beneficial since Arduino is one of the most popular platforms used in IoT development and implementation due to its compatibility with sensors and communication modules. Sai and Bhatia [8] surveyed the necessity of IoT security and the effectiveness of using cryptographic algorithms. This work involved a comparative analysis of IoT security models. The authors also investigated how using cryptography can reduce attacks through unauthorised access, tampering and data theft. Ramakrishna et al. [18] examined lightweight encryption methods using Raspberry Pi and compared the real-world performance of popular ciphers like HIGHT, SIMON, SPECK, Rectangle, Camellia, ITUbee, and AES.

Liu and Ning [19] discuss the tinyECC cryptographic library designed to incorporate the elliptic curve cryptography (ECC) technique for use in resource-constrained hardware associated with wireless sensor networks (WSNs). The Arduino library for tinyECC can be directly used on any Arduino board, including the classic UNO or the newer R4 boards. tinyECC incorporates assembly-level optimisations for critical operations, and its configurability makes it suitable for easy integration with most WSN applications. Tsao et al. [20] analysed AES-based encryption in IoT security, highlighting the algorithm’s non-proprietary and standardised symmetric encryption nature. Multiple AES implementations, including the tinyAES library, are discussed by the authors, and the tiny footprint of tinyAES, requiring limited resources to run, is appreciated. Iosifidis and Limniotis [21] explore integrating the Speck cipher into the Transport Layer Security (TLS) protocol, focusing on the performance gains when used in resource-constrained environments associated with IoT applications. Speck significantly improves the processing speed for IoT devices compared to AES-based encryption. The lightweight design of Speck makes it suitable for IoT deployments where the hardware limitations restrict the use of traditional ciphers. Santis et al. [22] look into the ChaCha20-Poly1305 (ChaChaPoly), which is an authenticated encryption algorithm that combines the ChaCha20 stream cipher and Poly1305 authenticator for high-performance applications in resource-constrained environments. ChaChaPoly utilises a combination of a key, nonce and counter and executes ARX (Add-Rotate-XOR) operations to encrypt the data. Shi and Guan [23] did a cryptanalysis study on the ACORN lightweight authenticated encryption algorithm. The algorithm operates through a multi-stage system, including initialisation, data processing, encryption, tag generation, and decryption. ACORN demonstrated robust security against forgery attacks, but demanded strict nonce management in deployments.

Khan et al. [24] evaluated the performance of the Ascon lightweight authenticated encryption algorithm in AI-enabled IoT devices. Ascon was standardised by NIST in 2023 for resource-constrained environments like IoT devices. Ascon is found to be efficient for IoT applications where balancing security with resource demands is critical. Table 1 shows the comparison of lightweight cryptographic algorithms popular in IoT applications [19,25,26,27,28,29,30]. Samotyja et al. in [31] investigated side-channel vulnerabilities in tinyECC. tinyECC is also found to be resource-intensive due to the elliptic curve operations, which are computationally intensive for resource-constrained devices. Matsukawa et al. found the probability of side-channel leakages in tinyAES [32]. The Speck algorithm was found to be susceptible to cryptanalytic attacks on reduced-round versions by Abed et al. [33]. Ascon and ACORN have been investigated for Fault Attacks vulnerabilities [34,35]. Ascon becomes complex if countermeasures against side-channel attacks are integrated into software using resource-constrained devices [36]. Side-channel attacks have been demonstrated against ACORN, highlighting the need for robust countermeasures [37]. ChaChaPoly requires proper nonce management, which could necessitate strict measures to prevent nonce reuse that could lead to security failures [38]. Although these algorithms are effective in various scenarios where constraints like limited computational ability, power requirement and memory requirement are not always prioritised, in situations where the overall footprint of the system is simple and limited, like a low-power edge device, there is a need for an algorithm that is optimised for all the limitations presented by the device.

Existing algorithms generally lack optimised performance for resource-constrained devices, as they fail to maintain a balance between security and performance, as shown in Table 2. While some algorithms, such as tinyAES, Speck, and Ascon, exhibit high-speed performance on Arduino-based hardware, their deterministic encryption methods compromise security. In contrast, ChaChaPoly and tinyECC offer enhanced security through non-deterministic encryption with cipher randomisation; however, this advantage is negated by their slower performance on resource-constrained hardware. Another critical factor for optimising sensor data encryption algorithms is transmission speed, which is particularly vital for low-power transmissions like LoRa, a preferred technology for sensor telemetry systems. The proposed ChaosFortress algorithm addresses these limitations by employing a non-deterministic encryption technique with computationally less intensive transformations. This method utilises three user-defined components: a nonce, a key, and a counter. The algorithm’s design prioritises high-speed performance on Arduino-based hardware. Furthermore, the ciphertext minimises transmission overhead, thereby facilitating very fast data transmission speeds.

The ChaosFortress library is a lightweight cryptographic solution designed specifically for resource-constrained environments such as the Internet of Things (IoT). At its core is a custom keystream generator employing a 32-bit key, a 32-bit nonce, and a 16-bit counter. The keystream itself is generated through a series of bitwise operations, including addition, shifting, rotation, and XOR. The design prioritises computational efficiency and a minimal memory footprint, ensuring a low CPU and RAM load suitable for low-power microcontrollers like those used in Arduino hardware. This efficiency contributes to rapid encryption and data transmission, further enhanced by optimised payload sizes. The algorithm is also optimised for floating-point inputs, reflecting its primary use case of processing sensor data. From a security perspective, ChaosFortress is engineered for resilience against common attacks targeting sensor data telemetry. It integrates cipher randomisation, which ensures that a unique ciphertext is generated even for identical plaintext inputs across separate encryption cycles. This combination of a small footprint, rapid execution, and robust security features makes the proposed algorithm a well-suited solution for securing lightweight applications.

## 3. Preliminaries

### 3.1. Hardware Setup

**Figure 2:**
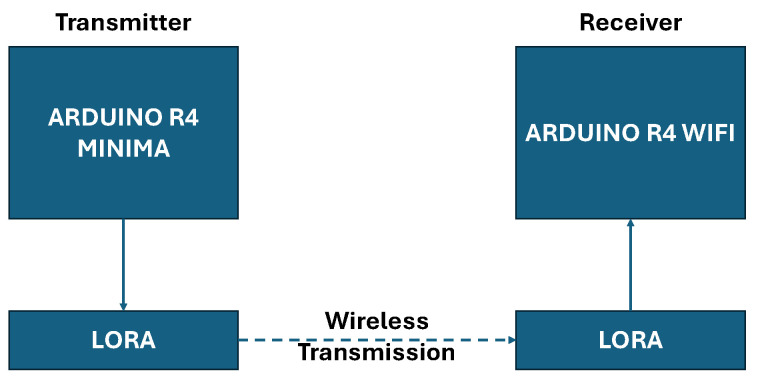
Architecture of the test setup.

The experimental validation and testing of the ChaosFortress cryptographic algorithm were done on the Arduino Platform [39]. The algorithm was ported into an Arduino library format, which can be used to program Arduino boards. Here, we chose the Arduino R4 boards(Arduino SRL, Turin, Italy), namely the Minima and the Wi-Fi. Arduino is also ideal from the comparative study perspective since the algorithms we compare with ChaosFortress are readily available as Arduino libraries [40]. Figure 2 shows the setup consisting of two modules. The first, a transmitter module, features an Arduino R4 Minima paired with a LoRa Hat, which receives data from a thermistor-based temperature sensor. The second module, the receiver, is based on an Arduino R4 Wi-Fi with a LoRa Hat. LoRa [41] was chosen for the comparative testing of our algorithm to make the validation more realistic with the real-world implementation of IoT systems. LoRa is one of the most popular wireless standards in IoT networks due to its low power consumption and long range, especially in metering of utilities like water, gas and electricity [42]. Among the available LoRa standards, we chose the 915–928 MHz frequency band following the AS923 standard [43]. A temperature sensor was added to the transmitter setup as the data source for all the testing. The sensor was a simple thermistor-based analog temperature sensor, which could be interfaced to the analog input pins of the Arduino and read using the built-in Analog to Digital Converter (ADC) [44]. The sensor is mounted to sense the ambient temperature or the temperature of any surface in contact with it. ADC converts the sensor’s analog signal to a digital form, making it compatible with the microcontroller. Arduino UNO R4 supports the 10-bit, 12-bit and 14-bit ADC resolutions. We use the standard 10-bit resolution, which converts the analog voltage output of the sensor into a digital value between 0 and 1023. This value is later converted into a temperature value in degrees Celsius using standard conversions for the thermistor-based sensor [45].

**Figure 3:**
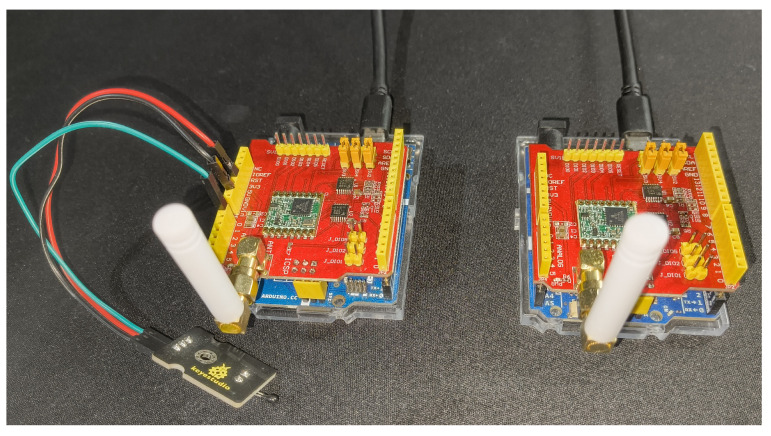
Arduino and LoRa modules used for testing.

Figure 3 shows the hardware modules used in the testing. The system for testing the ChaosFortress algorithm was designed using the Arduino UNO R4 boards, namely the Minima and Wi-Fi. These microcontroller boards based on the Renesas R4 microcontroller are considered the successor to the popular Arduino UNO board. They share the same form factor as the Arduino UNO and are compatible with the existing UNO ecosystem, with enhanced performance and features. The Renesas RA4M1 uses an ARM Cortex-M4 controller clocked at 48 MHz. This is a 32-bit microcontroller and supports 256 KB of flash memory and 32 KB of SRAM. The boards operate on a standard 5 V DC voltage and can take an input voltage from 6 to 24 V. Fourteen digital I/O pins and six analog input pins are available on the R4 boards, with an ADC that supports up to 14-bit and is tuned to 10-bit by default. These boards also support the standard communication interfaces like UART, SPI and I2C, which are essential for connecting sensors. The UNO form factor enables these boards to be compatible with most UNO shields, including the LoRa shield used for testing. The enhanced performance of the 32-bit Cortex-M4 core makes the R4 boards highly capable over the traditional Atmega328P controllers (Microchip Technology Inc., Chandler, AZ, USA) used in the classic UNO boards. The Arduino UNO R4 Wi-Fi incorporates an extra WI-Fi and Bluetooth feature using an add-on Espressif ESP32-S3 co-processor (Espressif Systems, Shanghai, China). This feature is useful when the boards are used as receivers, where the data needs to be updated into data management platforms using the internet. The LoRa shield was based on a LoRa SX1276 IC (Semtech Corporation, Camarillo, CA, USA), which operates at the 915–928 MHz frequency band [46]. The LoRa shield has a HAT-type architecture, which makes the connection between the LoRa and Arduino rigid and stable.

### 3.2. Data Security in Sensor Network

**Figure 4:**
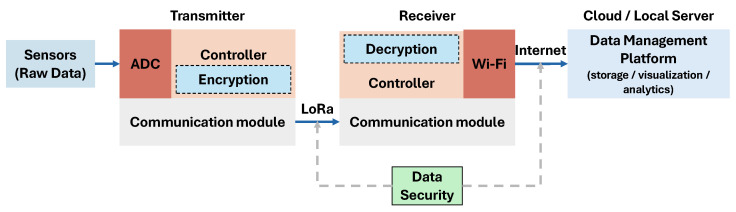
Data security in sensor networks.

The data security in a sensor network consists of two primary domains: local telemetry and internet communication. Local telemetry involves the point-to-point data transmission between transmitter and receiver modules. This stage utilises various wireless technologies to transfer sensor data before it is relayed to a central data management platform. For the purposes of this study, a LoRa wireless transmission system is employed for all local telemetry, and all subsequent testing and evaluations are based upon this specific implementation. Figure 4 shows the sensor network architecture and the need for data security in the data transmission. We are concerned with data security in local telemetry since we focus on lightweight encryption for resource-constrained devices in IoT systems.

The encryption of sensor data is a multi-stage process, beginning with data acquisition and concluding with secure decryption. Initially, the sensor generates raw data as an analog voltage relative to a 5 V DC reference. This signal is digitised using an Analog-to-Digital Converter (ADC). The resulting digital value is then converted into a calibrated temperature reading by applying transformation equations derived from the sensor’s official datasheet. This temperature value, constituting the plaintext, is subsequently encrypted using the ChaosFortress library’s encryption function. The resulting ciphertext is then transmitted as the payload via the LoRa protocol. Upon receipt, the gateway module decrypts the ciphertext using the decryption function within the ChaosFortress library, thereby recovering the original plaintext temperature value.

## 4. Methodology

### 4.1. ChaosFortress

The Arduino library developed to implement the ChaosFortress algorithm executes a multi-stage cryptographic process. This process can be categorised into four primary stages: key initialisation, keystream generation, encryption, and decryption. Key initialisation sets up the cryptographic state using the provided key and nonce. Subsequently, the keystream generation function produces a pseudorandom data stream. During encryption, this keystream is combined with the plaintext to generate the ciphertext. The decryption function, executed at the receiver, follows the same initialisation and keystream generation steps to reproduce the identical keystream, which is then used to reverse the encryption process and recover the original plaintext.

The ChaosFortress algorithm is designed to encrypt multiple data streams concurrently. The primary limitations on the number of simultaneous encryption processes are the physical input capacity of the microcontroller (i.e., the number of pins on an Arduino board) and its processing speed, as the total execution time scales linearly with each additional encryption operation. From an implementation standpoint, the algorithm is application-agnostic. Its design is not tailored to a specific data type or domain, making it universally applicable for any sensor data encryption task.

#### 4.1.1. Key Initialisation

The initialisation stage configures the cryptographic state of the ChaosFortress library. This process involves setting a 32-bit user-defined key (composed of two 16-bit high and low components), a 32-bit nonce, and a 16-bit counter, which is initialised to a default value of 0×0001. The nonce must be unique for each session to ensure encryption integrity, while the counter is incremented with each operation. This counter is fundamental to the library’s cipher randomisation feature, as its inclusion in the cryptographic operations ensures its non-deterministic encryption. This design, which balances carefully selected key and nonce sizes, maintains the algorithm’s lightweight characteristics without compromising security, a balance that has been validated by the NIST STS results.

#### 4.1.2. Keystream Generation

**Figure 5:**
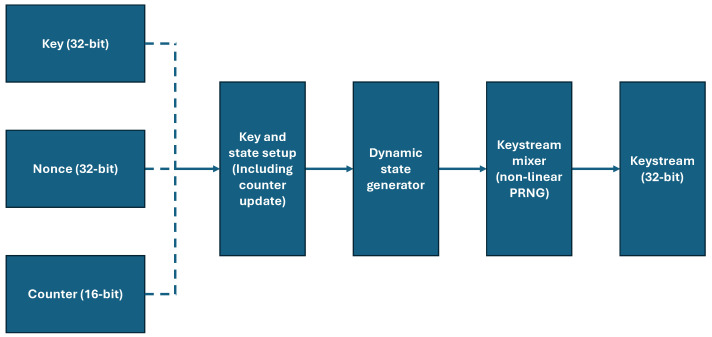
Keystream generation in ChaosFortress.

The keystream generation stage derives a unique keystream for each message or plaintext value using the key schedule and mixing of the user-defined components, as shown in Figure 5. The keystream generation process commences with a key and state setup stage, where the initial key, nonce, and counter are transformed through a series of Add-Rotate-XOR (ARX) operations. This initial mixing introduces statefulness and time-variant properties to the cryptographic process. Subsequently, a second round of similar operations evolves this internal state. This stage, however, operates on the intermediate state variables rather than the initial parameters, a method designed to inject non-linearity and entropy in a manner analogous to a chaotic random number generator. The multiple layers of bitwise operations contribute to the system’s overall cryptographic complexity. The resulting keystream is both message-unique and session-unique due to the respective functions of the counter and the nonce. The architecture emulates the principles of a stream cipher while remaining lightweight, ensuring its suitability for resource-constrained devices where performance and memory footprint are critical design parameters.

#### 4.1.3. Encryption

**Figure 6:**
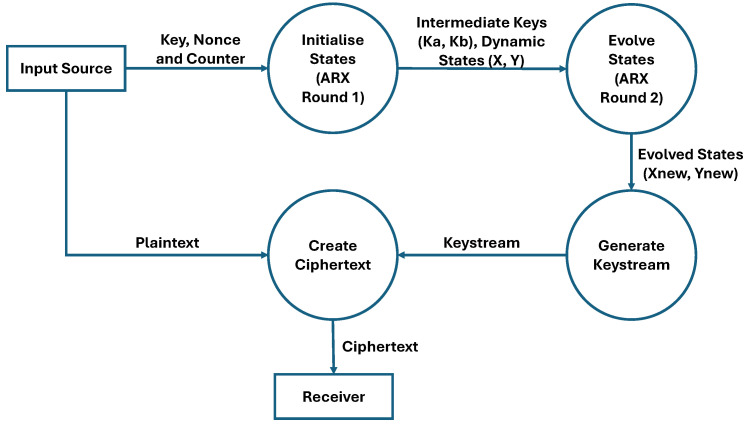
Data flow diagram of encryption operation.

**Figure 7:**
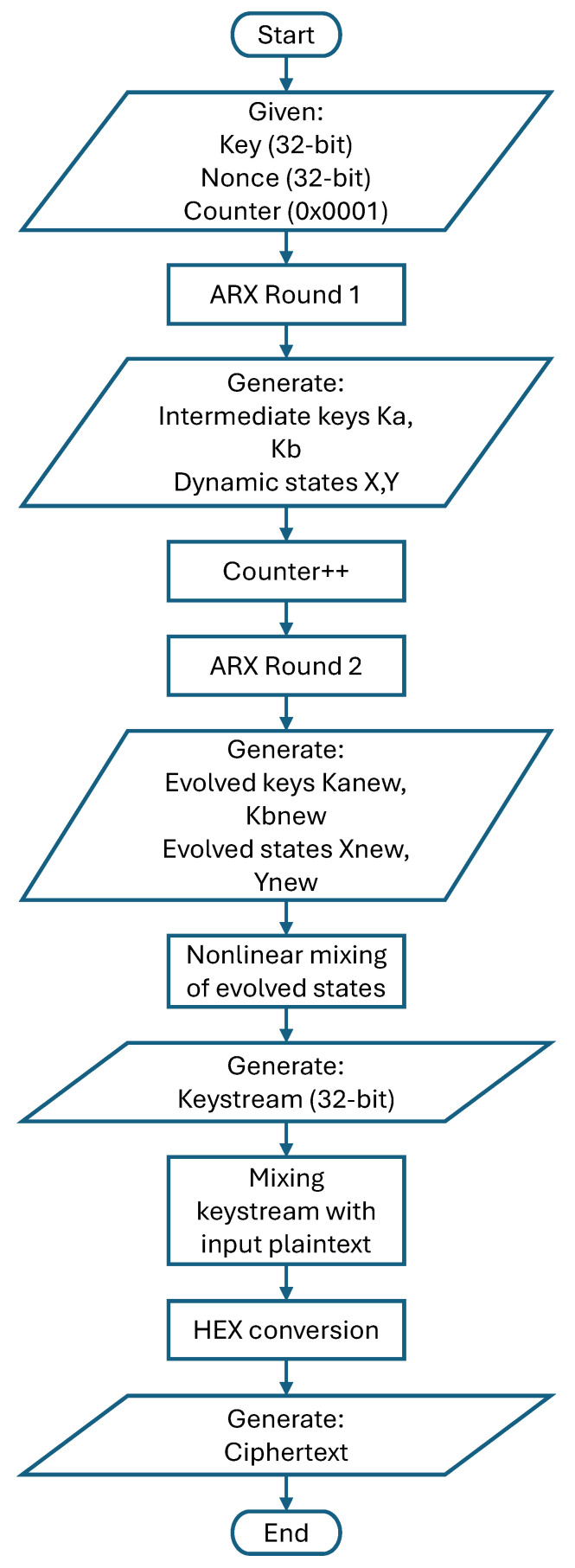
Flow chart of encryption operation.

During the encryption stage, the plaintext is transformed into ciphertext using the generated keystream, as depicted in the data flow diagram in Figure 6. The process is initialised with a user-defined 32-bit key, 32-bit nonce, and 16-bit counter. These inputs undergo the first round of Add-Rotate-XOR (ARX) operations to produce intermediate keys (Ka, Kb) and dynamic state variables (X, Y). Following this round, the counter is incremented. A second round of ARX operations then evolves the intermediate keys into new keys (Kanew, Kbnew) and further develops the dynamic state through additional mixing. This non-linear mixing of the evolved state variables efficiently scrambles the data to generate the final 32-bit keystream. This keystream is then combined with the plaintext, and the result is converted to a hexadecimal format to form the final ciphertext, which is then ready for transmission. The complete operational flowchart is illustrated in Figure 7.

#### 4.1.4. Decryption

The decrypt function of the ChaosFortress library reconstructs the original plaintext from the hex payload received. This step reverses the transformation undergone by the ciphertext and scales the value to recover the original information.

### 4.2. Implementation and Testing

The design of the ChaosFortress algorithm is engineered for resilience against many common attacks targeting Internet of Things (IoT) systems. Ciphertext-Only Attacks (COAs) do not work since the keystream is never reused. Since the keystream is unique per message because of cipher randomisation through nonce and counter combination, COA is ineffective on ChaosFortress encrypted data. The added nonlinearity through ARX operation adds to the immunity. Replay Attacks are prevented since the counter and nonce ensure each keystream is unique, and thus, when the message is replayed, the internal counter will have changed. The replayed ciphertext will not decrypt correctly unless the internal state is reverted and the counter is reset. A Chosen Plaintext Attack (CPA) is when the attacker knows the input. Since the keystream is never exposed directly, the data cannot be decrypted, and CPA cannot trivially break the encryption. A Key Guessing/Brute Force Attack is unlikely due to the overall application perspective. ChaosFortress integrates a combined entropy of 64 bits from the 32-bit key and 32-bit nonce. Although 64-bit is insufficient for high-security systems, brute force attacks are infeasible in a low-power IoT context, especially with varying counters. Code Injection or Fault Injection attacks cannot work since the library does not interpret the data structures or memory and only works with float values and HEX strings. Furthermore, the implementation avoids dynamic memory allocation and is designed to prevent buffer overflows, which systematically reduces the available attack surface.

## 5. Results

This section presents the test results of the in-house-developed ChaosFortress encryption algorithm, specifically designed for lightweight IoT applications. The experimental testbed comprised Arduino boards equipped with LoRa modules, forming a transmitter-receiver pair programmed for periodic data transmission. The hardware was programmed using the Arduino IDE, and data analysis was conducted using Python 3.10.11. A Linux virtual machine running on Oracle VirtualBox was employed to compile and execute the NIST STS for security analysis. At the same time, the native Linux terminal in the virtual machine was used to run NIST STS. The tests aimed to evaluate the effectiveness, reliability, robustness, and comparative performance of ChaosFortress. A range of popular encryption algorithms, including ACORN, Ascon, ChaChaPoly, Speck, tinyAES, and tinyECC, were assessed alongside ChaosFortress. ACORN, Ascon, ChaChaPoly, and Speck are part of the official Crypto library from NIST. The evaluation was based on four primary metrics: memory utilisation, execution time, transmission time and security analysis.

### 5.1. Memory Utlilisation

**Figure 8:**
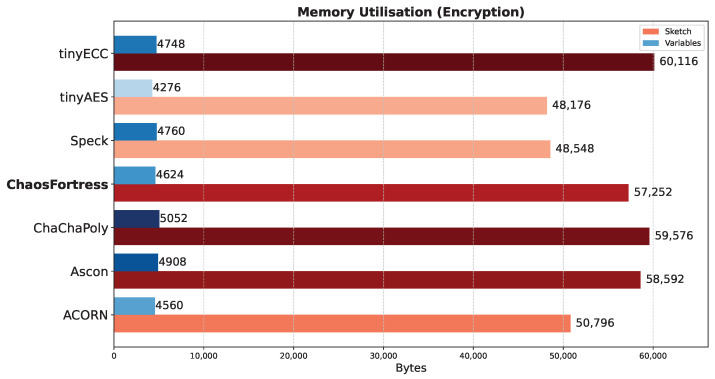
Memory use for encryption in the tested algorithms.

**Figure 9:**
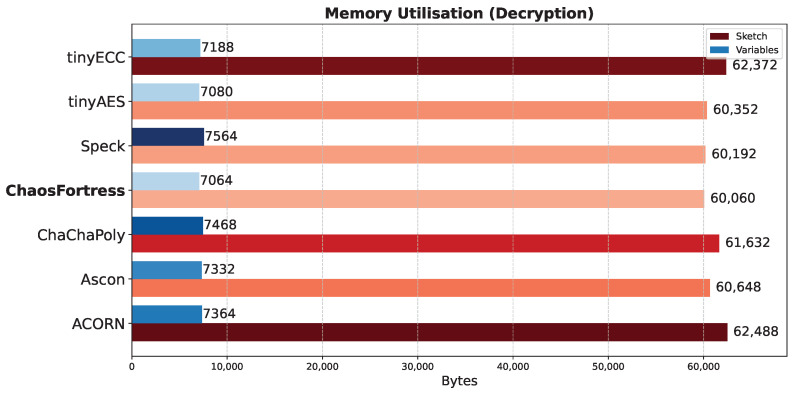
Memory use for decryption in the tested algorithms.

This test evaluated the memory consumption of each algorithm during identical encryption tasks. The experiment utilised an Arduino transmitter–receiver pair to transmit ciphertext generated by encrypting temperature values obtained from a temperature sensor. The analysis focused on the memory utilised for encryption in the transmitter and decryption in the receiver. The total memory consumed during encryption (Figure 8) or decryption (Figure 9) comprises the memory allocated to the Arduino code and the memory occupied by the global variables defined within the code. Generally, larger cryptographic libraries result in higher memory usage in the sketch. The memory used by the global variables remains relatively consistent across all algorithms, with only minor variations observed due to different keys, nonces, and other blocks involved in the encryption process. For encryption, ChaosFortress used 4624 bytes of memory for storing the variables and 57,252 bytes of memory for storing the code. These numbers were comparable to the tinyAES, which used the least memory for both variables and code at 4276 bytes and 48,176 bytes, respectively.

### 5.2. Execution Time

The execution time refers to the duration the Arduino program takes to encrypt the plaintext value obtained from the sensor into ciphertext. Faster encryption and decryption times are beneficial for enhancing operational speed and serve as an indicator of the algorithm’s efficiency, making it particularly advantageous for resource-constrained devices. This execution time is measured using the micros function, a feature natively available in Arduino. The micros function tracks the microseconds consumed by various scripts while running in a loop. Given that it relies on the internal clock of the Arduino board, it offers high accuracy, and its microsecond resolution facilitates clear distinctions in performance among different algorithms. Table 3 shows the execution time for the tested algorithms. ChaosFortress exhibited fast operations in both encryption and decryption, with 1155.09 microseconds for encryption and 230.13 microseconds for decryption, coming second only to Speck, which showed 211.40 microseconds for encryption and 124.40 microseconds for decryption. However, considering the deterministic nature of the Speck cipher, which yields the same ciphertext while encrypting messages with the same key, the non-deterministic nature of ChaosFortress offers enhanced security.

### 5.3. Transmission Time

**Figure 10:**
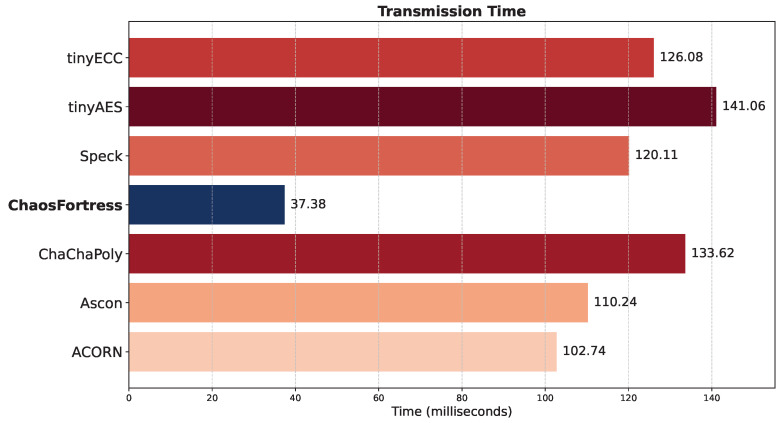
Transmission time for the tested algorithms.

The transmission time refers to the duration required by a transmitter–receiver pair to complete one payload transmission successfully. This measurement encompasses the time the payload travels from the transmitter to the receiver. The transmission time was determined experimentally by using the transmitter–receiver pair to send multiple transmissions of inputs formatted as float temperature values from the thermistor. Time stamps, recorded with millisecond resolution on both the transmitter and receiver modules, were used to calculate the transmission time, as shown in Figure 10. ChaosFortress outperformed the other algorithms in the transmission tests with a good margin. When ChaosFortress took 37.38 ms on average to complete a successful transmission from the transmitter to the receiver using LoRa, the runner-up, ACORN, took 102.74 ms, which is three times slower than ChaosFortress.

### 5.4. Security Analysis

To assess the security of the ChaosFortress algorithm, a comparative analysis was performed using the National Institute of Standards and Technology Statistical Test Suite (NIST STS). This suite is a well-established benchmark for evaluating the randomness of a cipher’s output, a critical indicator of its cryptographic security. In lightweight cryptography, the ability of the algorithm to generate statistically random outputs determines the level of security it can provide. NIST STS evaluates this level of randomness and thus is an ideal tool for evaluating the algorithm’s effectiveness. The results of the NIST STS are based on the *p*-values generated by the test. *p*-values serve as a statistical metric to assess the randomness of a data sequence. They quantify the extent to which the sequence adheres to perfect randomness, thereby providing a measure of its quality. Each statistical test run in the STS computes the probabilities of the test statistic, quantifying some feature of randomness. These features include tests for checking whether the number of zeroes and ones is balanced, checking for repetitive patterns, measuring pattern complexity and the sequence’s unpredictability. In short, the *p*-value in NIST STS is the probability that a test result is consistent with randomness. A *p*-value close to 1 appears to be random, and a *p*-value close to 0 indicates the unlikeliness of a random source. A good cryptographic algorithm is expected to produce high and uniformly distributed *p*-values across all tests. NIST STS is also a crucial early means to identify a weak design in an encryption algorithm.

**Figure 11:**
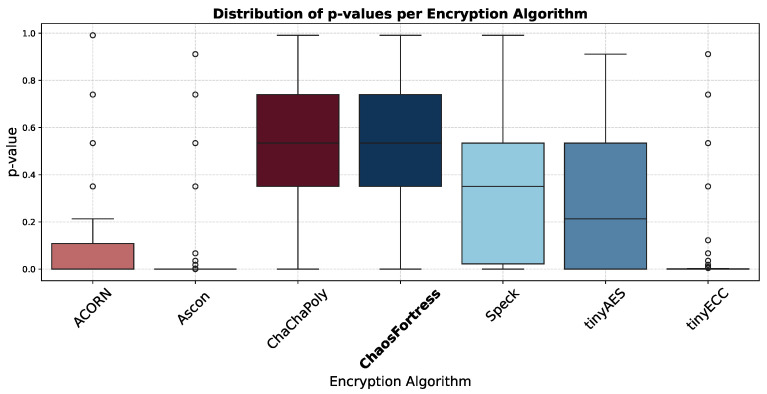
Box plot of *p*-value distribution in the tested algorithms.

The results of STS can be visualised in many ways. The box plot in Figure 11 shows the distribution of *p*-values across various algorithms in different tests: a median value close to 0.5 shows ideal randomness, and a narrow box points towards consistent randomness. *p*-values above 0.01 are considered a pass, but higher values are preferred for better performance.

**Figure 12:**
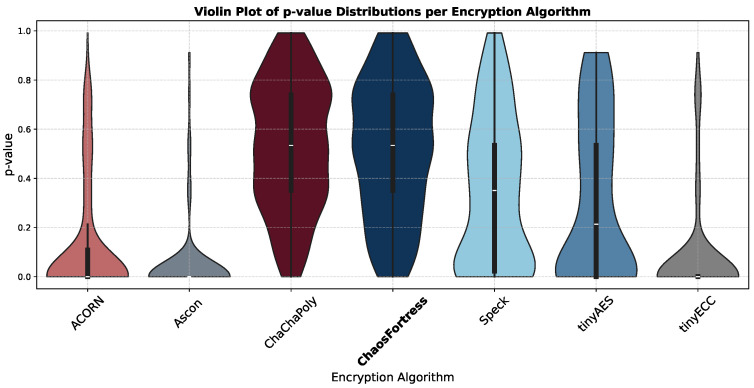
Violin plot of *p*-values for the tested algorithms.

Another key method to visualise STS results is using a violin plot, similar to a box plot but using a rotated KDE (Kernel Density Estimation) on each side, showing the density of the *p*-values. It gives the range and the concentration locations of *p*-values, as shown in Figure 12.

**Figure 13:**
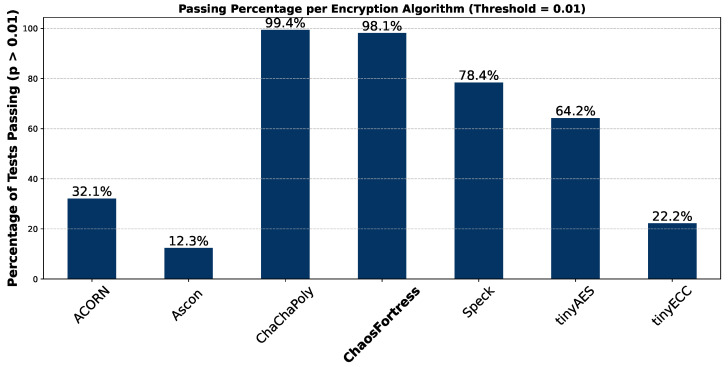
Pass percentage of the tested algorithms in NIST STS.

Figure 13 shows a bar graph depicting the pass percentage per algorithm in the context of NIST STS testing. It indicates how well each algorithm performed across the statistical randomness tests. A threshold or significance level of 0.01 was set as standard in the test to differentiate the algorithms’ performance. The pass percentage of each algorithm compares the algorithms side by side and identifies which is likely to produce non-random and predictable outputs. Algorithms with high pass rates are statistically random enough for cryptographic implementation.

## 6. Discussion

Across the lightweight cryptographic algorithms tested, memory utilisation showed very small differences, making it the least significant aspect of the comparative analysis. However, execution time showed significant differences across the algorithms. tinyECC had a longer exponential execution time both for encryption and decryption, making it not an ideal choice for IoT security. tinyECC was found to be the most resource-intensive algorithm among all those tested. In encryption tasks, it required execution times approximately 200 times longer than ACORN, the next slowest algorithm. For decryption, it was about 100 times slower than tinyAES, the second slowest in that category. Such performance overhead makes tinyECC highly impractical for resource-constrained IoT devices. Among the remaining algorithms, Speck and ChaosFortress demonstrated superior performance in both encryption and decryption. While Speck achieved the highest overall speed, ChaosFortress offered a strong balance between speed and security, making it a competitive alternative for IoT applications. ChaosFortress, ranking as the second fastest overall, surpassed ChaChaPoly by a factor of three in encryption speed and Ascon by a factor of eight in decryption. These results highlight ChaosFortress’s ability to deliver consistently high performance across both operations, positioning it as a strong all-round choice despite not being the single fastest in either category.

In the evaluation of transmission time, ChaosFortress demonstrated the fastest performance of all algorithms evaluated, executing three times more quickly than the nearest alternative, ACORN. This superior speed is particularly advantageous in the context of Internet of Things (IoT) sensor networks, where bandwidth and data rates are typically constrained. Furthermore, reduced transmission latency leads to more rapid feedback within a system, which consequently enhances its overall operational performance.

A key differentiating feature, cipher randomisation, was supported in only three of the seven algorithms. Cipher randomisation is defined as the ability of a cryptographic algorithm to produce a different ciphertext for the same plaintext input upon each execution. This capability substantially strengthens the encryption against pattern attacks and plaintext attacks. Although ChaosFortress, ChaChaPoly, and tinyECC all exhibited cipher randomisation, the slower execution speed of tinyECC rendered it less viable for this application in comparison to the other two.

**Figure 14:**
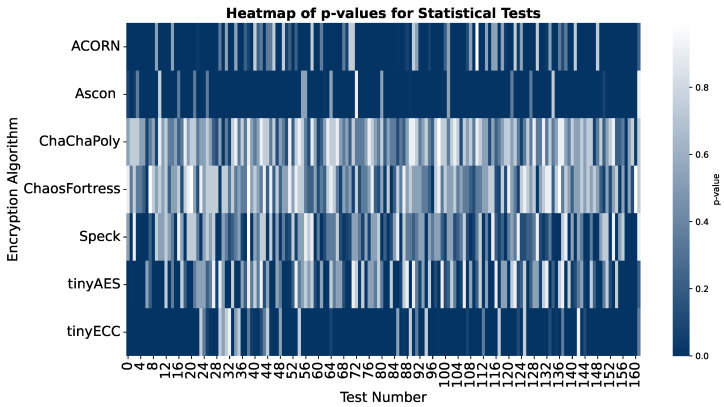
Overall heatmap of *p*-values for the tested algorithms across 162 tests in NIST STS.

In the security analysis conducted using the National Institute of Standards and Technology Statistical Test Suite (NIST STS), significant variations in the security performance of the algorithms were revealed. The boxplot in Figure 11 illustrates that ChaChaPoly and ChaosFortress demonstrated superior performance, yielding *p*-values that were uniformly distributed across the testing suite. This uniformity is a key indicator of high-quality cryptographic randomness. Conversely, tinyECC and Ascon yielded the least favourable results. This finding is further validated by the violin plot in Figure 12, which depicts the data’s density distribution. The distributions for ChaChaPoly and ChaosFortress are visibly even and wide, in stark contrast to the localised and non-uniform distributions observed for the other algorithms. This suggests that the latter group of algorithms exhibit weaker randomisation properties. The pass or fail threshold for each test within the NIST STS suite is a value between 0 and 1. As illustrated in Figure 14, which presents the pass rate, ChaChaPoly and ChaosFortress significantly outperformed the other algorithms, achieving scores of 99.4% and 98.1%, respectively. In contrast, Ascon demonstrated the weakest performance, followed by tinyECC. Table 4 shows the overall performance comparison of ChaosFortress with the existing algorithms in the various key aspects.

**Figure 15:**
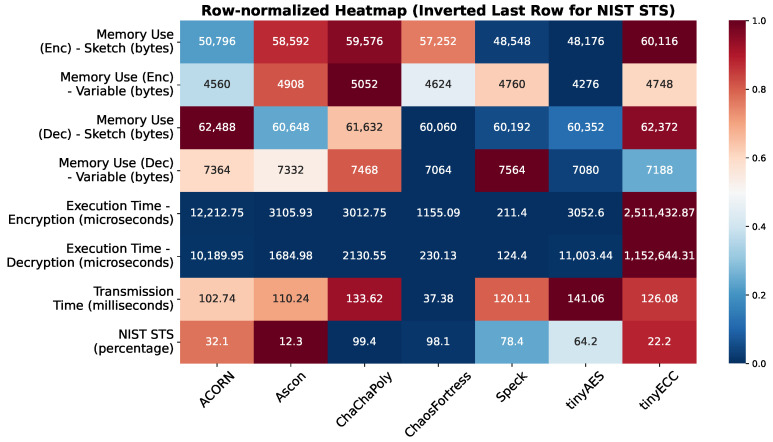
Performance map of the tested algorithms.

A heatmap of the *p*-values from all 162 statistical tests affirms the dominance of ChaChaPoly and ChaosFortress. The performance map in Figure 15 provides a holistic comparison of all algorithms, considering various performance metrics. This analysis highlights the specific advantages of ChaosFortress for implementations of lightweight cryptography, particularly in resource-constrained applications where efficiency is paramount.

While the 64-bit key and nonce combination of ChaosFortress offers a lower theoretical security level than the 128/256-bit configurations, this trade-off is pivotal to achieving its computationally efficient architecture. This design prioritises low computational overhead, a critical requirement for resource-constrained applications. The viability of this approach is supported by the algorithm’s successful performance in the NIST STS evaluation, which indicates that its practical security is sufficient for the intended use cases. Furthermore, the algorithm possesses inherent flexibility, as it can be adapted to use 128-bit or 256-bit encryption by increasing the key and nonce sizes. Such a modification would cater for sensitive applications demanding higher security, although it would consequently necessitate more resources.

## 7. Conclusions

ChaosFortress demonstrated consistent performance across all tests. Memory usage was comparable among all the algorithms, with no clear advantage for any one of them. It showed a clear advantage in transmission tests, achieving speeds three times faster than the second best algorithm. Although Speck was slightly faster in execution time, its deterministic design leaves it highly vulnerable to replay and ciphertext-only attacks. Speck’s lower performance in the NIST Statistical Test Suite (STS) ranked it behind ChaosFortress, which delivered standout results in timing tests. ChaChaPoly achieved a slight lead in the STS (approximately 1.3%), but its slower timing performance gave ChaosFortress the overall advantage. Overall, when assessed across memory utilisation, execution time, transmission time, and security, the in-house developed ChaosFortress emerged as the top choice among the lightweight cryptographic algorithms tested. Future studies could build on this work by exploring the use of larger key sizes and a nonce to enhance encryption strength. Future work could optimise the computational efficiency of ChaosFortress while preserving its lightweight nature. Integrating lightweight cryptography into communication protocols like LoRa could support built-in encryption alongside modulation.

## Figures and Tables

**Table 1:** Comparison of existing lightweight cryptographic encryption algorithms.

Algorithm	Type	Security Level	Speed on Arduino	Cipher Randomisation
tinyECC [19]	Public-Key (ECC)	128/256-bit	slow	yes
tinyAES [20]	Symmetric Block Cipher	128-bit	fast	no
Speck(NIST) [21]	Symmetric Block Cipher	128-bit	very fast	no
ChaChaPoly [22]	Stream + MAC (AEAD)	128/256-bit	moderate (slower than AES)	yes
ACORN [23]	Stream Cipher (AEAD)	128-bit	moderate	no
Ascon [24]	Authenticated Cipher	128-bit	fast on 32-bit MCUs	no

**Table 2:** Strengths and weaknesses of existing algorithms.

Algorithm	Strengths	Weaknesses
tinyECC	smaller keys, suitable for key exchange and digital signature	computationally intensive, high memory footprint and energy usage
tinyAES	widely adopted and standardised, small footprint	vulnerable to side-channel attacks, slower than stream ciphers
Speck(NIST)	extremely lightweight, fast, small footprint	less secure, known differential and linear attacks
ChaChaPoly	string securty, built-in authentication	large memory footprint, less efficient in low power hardware
ACORN	low energy usage, built-in authentication	slower than some block ciphers
Ascon	balanced for speed and security, resistance to side-channel attacks	not optimized for high-speed bulk encryption

**Table 3:** Execution time for encryption and decryption in tested algorithms.

Algorithm	Encryption (ms)	Decryption (ms)
tinyECC	2,511,432.87	1,152,644.31
tinyAES	3052.60	11,003.44
Speck	211.40	124.40
Ascon	3105.93	1684.98
ChaosFortress	1155.09	230.13
ChaChaPoly	3012.75	2130.55
ACORN	12,212.75	10,189.95

**Table 4:** Performance comparison of ChaosFortress with existing algorithms.

Algorithm	Security Level	Execution Speed	Transmission Speed	Cipher Randomisation	NIST STS Performance
ACORN	128-bit	moderate	fast	no	low
Ascon	128-bit	fast on 32-bit MCUs	fast	no	very low
ChaChaPoly	128/256-bit	moderate	slow	yes	very high
ChaosFortress	64-bit	very fast	very fast	yes	very high
Speck(NIST)	128-bit	very fast	fast	no	moderate
tinyAES	128-bit	fast	very slow	no	moderate
tinyECC	128/256-bit	slow	slow	yes	very low

## Data Availability

Data not available publicly and is maintained privately at Curtin University, Australia.

## Abbreviations

The following abbreviations are used in this manuscript:

ACORN	Additive Congruential Random Number generator
ADC	Analog to Digital Converter
AEAD	Authenticated Encryption with Associated Data
AES	Advanced Encryption Standard
API	Application Programming Interface
ARM	Advanced RISC Machines
ARX	Add-Rotate-XOR
COA	Ciphertext-Only Attacks
CPA	Chosen Plaintext Attacks
ECC	Elliptic-Curve Cryptography
HIGHT	HIGh security and light weigHT
I2C	Inter-Integrated Circuit
IoT	Internet of Things
KDE	Kernel Density Estimation
LoRa	Long Range
NIST	National Institute of Standards and Technology
PRNG	Pseudorandom Number Generator
RFID	Radio Frequency Identification
SPI	Serial Peripheral Interface
SRAM	Static Random Access Memory
UART	Universal Asynchronous Receiver/ Transmitter
WDN	Water Distribution Network
WSN	Wireless Sensor Network
XOR	Exclusive OR
